# Prognosis and treatment of FOLFOX therapy related interstitial pneumonia: a plea for multimodal immune modulating therapy in the respiratory insufficient patient

**DOI:** 10.1186/s12885-017-3576-y

**Published:** 2017-08-29

**Authors:** Annick De Weerdt, Amélie Dendooven, Annemie Snoeckx, Jan Pen, Martin Lammens, Philippe G. Jorens

**Affiliations:** 1 Department of Intensive Care Medicine, Antwerp University Hospital, University of Antwerp, Wilrijkstraat 10, 2650 Edegem, Belgium; 2 Department of Pathology, Antwerp University Hospital, University of Antwerp, Edegem, Belgium; 3 Department of Radiology, Antwerp University Hospital, University of Antwerp, Edegem, Belgium; 4 Department of Gastroenterology, Heilig Hart Hospital, Lier, Belgium

**Keywords:** FOLFOX, Oxaliplatin toxicity, Chemotherapy lung, Interstitial lung disease, Interstitial pneumonia, Drug induced pulmonary toxicity, Immune globulins, Cyclophosphamide, Case report and review

## Abstract

**Background:**

The FOLFOX regimen, i.e., folinic acid (FOL), fluorouracil (F) and oxaliplatin (OX), is a drug cocktail that is used to treat gastric and colorectal cancers. Despite the concomitant improvements in response rate, duration of response and patient survival, reports of serious toxic pulmonary side effects have progressively emerged.

**Case presentation:**

We describe a patient who was treated with FOLFOX as an adjuvant to a rectosigmoidal resection of a rectosigmoidal carcinoma and who developed respiratory insufficiency requiring mechanical ventilation. Computed tomography (CT) imaging and open lung biopsy findings were compatible with interstitial pneumonia (IP). She received multimodal combination treatment (acetylcysteine, corticosteroids, immune globulins and cyclophosphamide) and survived.

We performed a systematic literature search and reviewed all 45 reported cases of FOLFOX-related lung toxicity and/or pulmonary fibrosis for their clinical characteristics and their outcomes related to therapy.

**Conclusions:**

We found that for the 45 cases with available data, the median age was 70 years, and the male–female ratio was 3.5: 1. In the patients exhibiting only mild respiratory symptoms, discontinuation of the culprit drug (oxaliplatin) resulted in a 100% regression of the symptoms. However the prognosis of the respiratory insufficient patient proved to be grim: death occurred in 76.9% of the cases despite conventional treatment with corticosteroids. We therefore urge oncologists and critical care specialists not to limit their interventions to the discontinuation of chemotherapy, artificial ventilation, corticosteroids and glutathione replenishment and to consider the gradual introduction of additional immune-modulating agents whenever life-threatening respiratory symptoms in oxaliplatin-treated patients do not subside; all the more so considering the fact that our analysis showed that every patient who survived intubation and mechanical ventilation experienced a full clinical recovery.

## Background

Since the middle of the previous century, 5-fluorouracil (5-FU) has been the cornerstone in the treatment of colorectal cancer. The initial addition of folinic acid (leucovorin) and subsequent addition of oxaliplatin (OX) resulted in improved response rates, longer remissions and an increase in patient survival [1, 2]. Subsequently, the combination of folinic acid, 5-fluorouracil and oxaliplatin, i.e., the so-called FOLFOX regimen, became a well-established treatment for colorectal malignancy either as in monotherapy or as an adjuvant to surgery [1]. With the widespread use of this triple chemotherapeutic combination therapy, reports of increased toxicity (e.g., peripheral neuropathy, neutropenia, thrombocytopenia, vomiting) compared with the 5-FU/leucovorin treatment emerged [3]. Additionally, interstitial lung disease has been reported. In the majority of cases, the noxious pulmonary effects occur rather late in the course of therapy [4–9], although there have been exceptions to this rule [10–14]. Although pulmonary toxicity in conjunction with FOLFOX therapy is uncommon (≤ 1.5%) [15], it can be lethal despite the immediate discontinuation of the chemotherapeutic drugs and the initiation of immunotherapy (i.e., corticosteroids). Indirect arguments designating oxaliplatin as the causative pulmonary toxic agent can be found in more than one publication [4, 7, 12, 14, 16–19].

We describe a patient with profound pulmonary toxicity secondary to FOLFOX who was successfully treated with a combination of acetylcysteine, corticosteroids, immune globulins and cyclophosphamide. Additionally we reviewed all other reports of similar cases.

## Case presentation

**Fig. 1:**
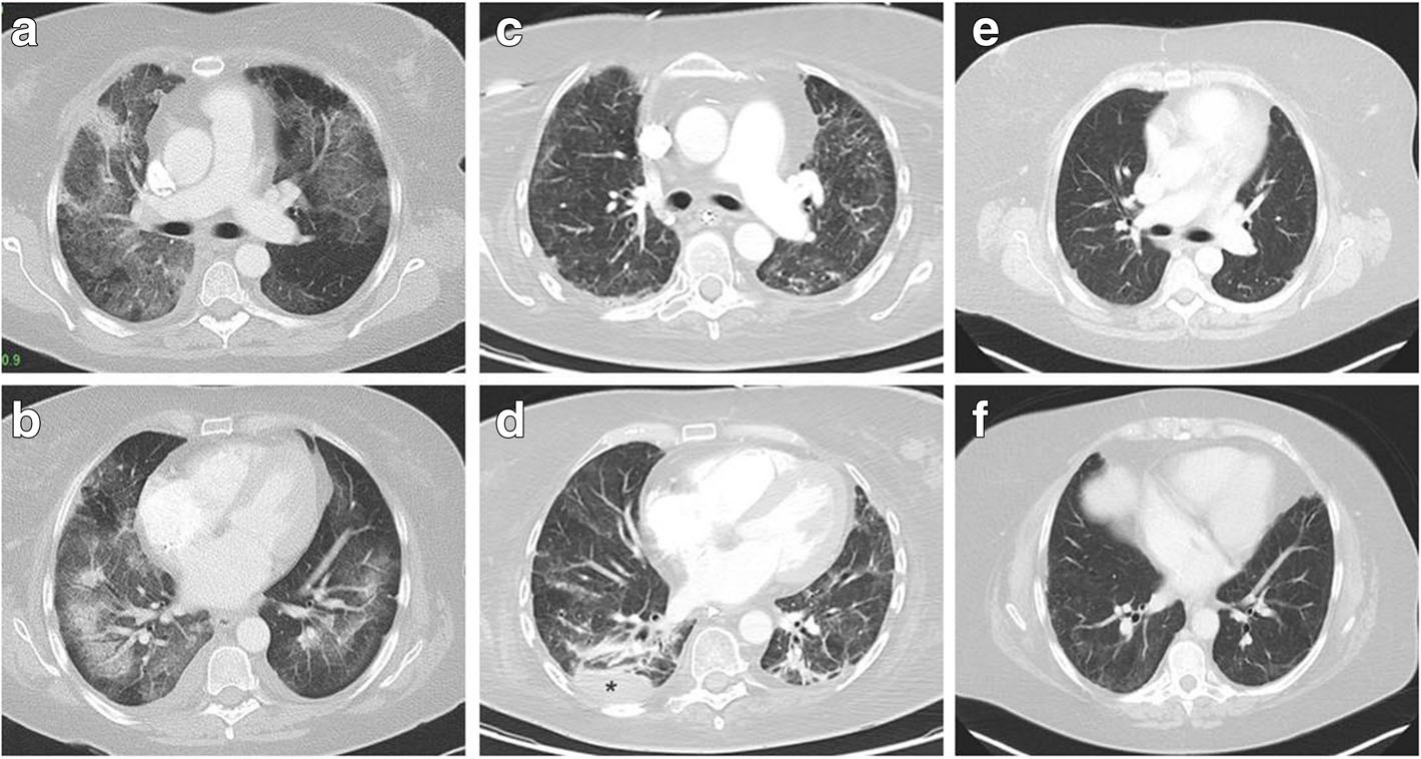
axial CT images of the chest in the lung window setting at the levels of the upper lobes (images in a, c and e) and the lower lobes (images in b, d and f). CT examination at the time of diagnosis revealed extensive abnormalities in both lungs with diffuse ground glass abnormalities in both the upper (**a**) and lower lobes (**b**). Associated areas of consolidation at the lower lobe level were present. Follow-up images 11 weeks after multimodal therapy for ILD revealed a good resolution of the ground glass abnormalities and consolidation with only minor parenchymal changes in the upper lobes, some small foci of ground glass abnormalities and some parenchymal bands (**c**). In the lower lobes, the nodular area (**d** asterisk) was consistent with loculated postoperative fluid due to the open-lung biopsy. The most recent examination more than 4 years after the event (**e, f**) showed no abnormalities

A 49-year-old non-smoking female patient, who received a diagnosis of rectosigmoidal carcinoma 4 months prior and was treated with (laparoscopic) rectosigmoidal resection and adjuvant FOLFOX chemotherapy, was admitted to the hospital due to progressive dyspnoea approximately 3 weeks after the sixth chemotherapy session. CT examination of the lungs revealed extensive abnormalities in both lungs with diffuse ground glass abnormalities in both the upper (Fig. 1a) and lower lobes (Fig. 1b) and areas of consolidation at the level of the lower lobes (Fig. 1b). Antibiotics were empirically prescribed (moxifloxacin) from day one. Hypoxic respiratory insufficiency arose and necessitated intubation 1 week after admission. Immediately thereafter, she was referred to our university hospital. On admission to our intensive care unit (ICU), 70% oxygen (PEEP 8 cm H_2_0) was needed to prevent frank hypoxemia. The serum white blood cell count amounted to 19 × 10 ^9^/L (the normal value is up to 10 × 10 ^9^/L). Predominantly, neutrophils (78.8%, 14.97 × 10 ^9^/L) and to a lesser extent lymphocytes (16.3%, 3.10 × 10 ^9^/L), were present. There was no peripheral eosinophilia. The C-reactive protein (CRP) level was low (1.2 mg/dl), and there were no biochemical signs of other organ failure (i.e., serum creatinine 0.55 mg/dl).

Shortly after admittance to our ICU, a bronchoalveolar lavage (BAL) was performed in the right middle lobe and revealed a white blood cell count of 44/m^3^ that mostly consisted of neutrophils (90%) in addition to 4% lymphocytes, 6% macrophages and no eosinophils, which suggested infectious lung disease or acute diffuse lung injury [20]. However, no microorganisms (e.g., (myco)bacteria, moulds, fungi, or viruses (entero-, rhino-, parainfluenza, adeno-, herpes simplex, or cytomegalovirus)) were detected. PCR on the BAL fluid for herpes simplex, cytomegalovirus, Epstein-Barr virus, Chlamydia pneumoniae, Mycoplasma pneumoniae and Bordetella pertussis also proved negative. Additional histopathological examination of the lavage liquid did not reveal malignant cells. There was no family history of interstitial lung disease (ILD), and there were no coexisting medical conditions that favoured the development of ILD. Moreover, there had been no occupational exposure to pulmonary toxins and no prior use of potential ILD-causing drugs (e.g., bleomycin, busulphan, gemcitabine, mitomycin, paclitaxel, docetaxel, nitrofurantoin, amiodarone), with the exception of the FOLFOX chemotherapy.

Given the proof of the absence of pulmonary infection, at 48 h after admission, high-dose corticosteroids (methylprednisolone 4 × 250 mg per day) were administered intravenously (IV) over 5 consecutive days followed by a tapering scheme. This therapy was initiated while considering the possibility of an autoimmune disease or chemotherapy-related pulmonary toxicity [21]. Meanwhile, IV acetylcysteine (1800 mg per day) had been administered from day one in our hospital with the initial intention of preventing contrast-induced nephropathy in this CT-scanned patient.

In view of the fact that screening for autoimmune and systemic diseases (e.g., anti-nuclear antibodies, anti-neutrophil cytoplasmatic antibodies, complement factors, circulating immune complexes, lupus anticoagulant, immune globulin dosage, retinal fundoscopy) revealed no aberrations, and based on the absence of a favourable respiratory evolution after 14 days of therapy, a surgical (open) lung biopsy (right middle lobe) and a tracheotomy were performed.

**Fig. 2:**
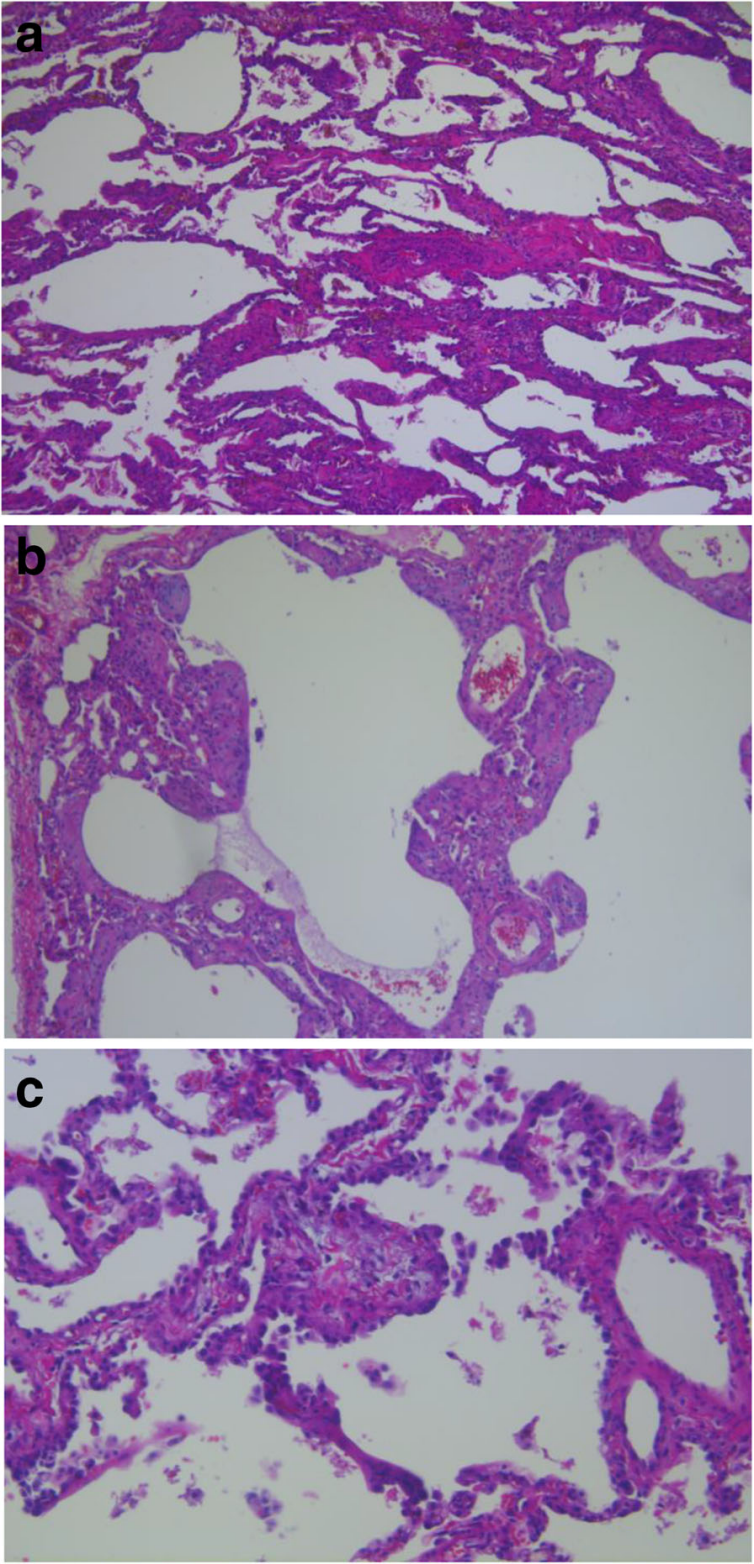
lung tissue (day 14, open-lung biopsy, staining Hematoxylin Eosin) exhibiting a pattern compatible with on-going damage of the alveolar epithelium with evolving pulmonary fibrosis. **a** magnification 37×: histology (wedge biopsy) revealing lung tissue with a disturbed architecture. The alveolar septa are thickened in a non-specific interstitial pneumonia (NSIP) pattern with lymphocytic inflammatory infiltrate and fibrosis. **b** magnification 185×: the alveolar lumina exhibit the presence of an exudate. **c** Magnification 380×: reactive cuboid pneumocytes line the alveolar lumina. This picture is consistent with interstitial pneumonitis

An initial quick examination of the lung biopsy fragments revealed an interstitial pattern of disease with widening of the alveolar septa. While awaiting further microscopic characterization, immune globulins (Sandoglobulin 0.4 g/kg) were administered intravenously during a five-day period in an attempt to reduce the pulmonary inflammation that led to fibrosis [22–24]. Approximately a fortnight later, a single dose of cyclophosphamide (10.5 mg/kg = 1000 mg, Endoxan, Baxter, Braine-l-Alleud Walloon Brabant, Lessines Hainaut, Belgium) was administered due to the persistent need for ventilatory support and the possibility of an unspecified immunological process. The intravenous administration of acetylcysteine was continued. After these therapeutic interventions, the oxygen demand gradually fell. The definite histopathological findings validated the presence of on-going damage of the alveolar epithelium with evolving pulmonary fibrosis. Thickened alveolar septa with lymphocytic inflammatory infiltrate and fibrosis and an exudate in the alveolar lumina lined with reactive cuboid pneumocytes were present. There were no arguments for concomitant vasculitis, infection or malignancy (Fig. 2).

The interstitial pattern on chest X-ray and follow-up CT gradually dissolved. A follow-up CT-scan 13 weeks after admittance to our ICU revealed a good resolution of the ground glass abnormalities and consolidation with only minor residual parenchymal changes in the upper lobes (Fig. 1c).

The patient remained dependent upon mechanical ventilation until day 80 due to intercurrent ventilator-associated pulmonary infections that were treated with broad-spectrum antibiotics, acute bilateral pulmonary embolism, critical illness myopathy (secondary to the glucocorticoid treatment) and polyneuropathy (electromyographically confirmed), all of which contributed to the general neuromuscular weakness and failure to wean from mechanical ventilation.

On day 101, she was referred to the department of respiratory medicine for further care still receiving oral methylprednisolone at a dosage of 20 mg per day and acetylcysteine in a daily dosage of 1200 mg.

She left the hospital 8 months after admission and resumed work 1 year after discharge. She remained under medical supervision and, in a diagnostic work up for evolving carcinoembryonic antigen, underwent computed tomography of the lungs more than 4 years later. No residual pulmonary abnormalities were found (Fig. 1 e-f).

## Methods

A systematic literature search was performed in PubMed for all publications (case reports and case series) regarding FOLFOX-related pulmonary toxicity with or without pulmonary fibrosis, respiratory insufficiency, intubation and artificial ventilation. To reduce/eliminate the chance of bias, we excluded all reported cases that were not treated solely with FOLFOX (e.g., FOLFOX/bevacizumab) or not solely attributable to FOLFOX. The corresponding authors of the reports that did not mention the ventilation or intubation statuses of their patients were contacted by e-mail or telephone to provide this information because it was our aim to describe and compare treatment strategies in the dramatic cases, i.e., the respiratory insufficient, intubated patients. Forty-five cases were identified [4–19, 25–39]. The data were analyzed using Student’s t-tests. Values of *p* < 0.05 were considered statistically significant.

## Results

Interstitial lung disease associated with FOLFOX therapy seems to be a global problem. Physicians on nearly every continent (Table 1) have published reports regarding this topic. Including our own, there are currently 45 reports about FOLFOX-related pulmonary toxicity, including 18 from Asia, 16 from Europe, 5 from North America, 3 from South America and 3 from Oceania.

**Table 1:** Overview of all reported cases of FOLFOX therapy related pulmonary toxicity

Reference	Patient gender/age (years)	Oncologic diagnosis	Number of FOLFOX cycles	Total dose OX (mg/m2)	Presumed lung disease (Radiology/Laboratory)	Pathology	ILD treatment other than discontinuation of FOLFOX	Artificial Ventilation	Outcome
2001 Trisolini Italy	M/60	Colorectal cancer	7	910	ILD	BAL: DAD cells TBB: fibroblastic plugs in alveolar spaces, DAD cells	Corticosteroids	No	Complete resolution
2002 Gagnadoux France	F/60	Colorectal cancer	8	680	EP	TBB: no abnormalities	None	No	Complete resolution
2006 Ruiz-Casado Spain	M/67	Hepatocellular cancer	NA	1100	PF	NA	None	No	Improvement
2005 Hernandez Yagüe Spain	F/68	Colorectal cancer	6	510	PF	LB: IP + DAD	Corticosteroids	Yes	Death
2006 Jung Korea	M/64	Gastric cancer	2	200	PF	TBB: organizing pneumonia	Corticosteroids	No	Improvement
2006 Jung Korea	M/75	Gastric cancer	1	100	ILD	TBB: DAD with hyaline membranes	Corticosteroids	No	Improvement
2006 Pasetto Italy	M/74	Colorectal cancer	6	510	ARDS	TBB: no infection	Corticosteroids	NA	Death
2007 Garrido Chile	F/30	Colorectal cancer	6	510	ILD	LB: COP	Corticosteroids	No	Complete resolution
2008 Wilcox USA	M/71	Colorectal cancer	6	600	ILD	NA	Corticosteroids	Yes	Death
2008 Wilcox USA	F/77	Colorectal cancer	12	1200	IP	NA	None	No	Improvement
2008 Wilcox USA	M/69	Colorectal cancer	6	NA	ILD	TBB: COP	Acetylcysteine Corticosteroids	No	Improvement
2007 Mundt Germany	M/66	Colorectal cancer	12	1020	Infectious pneumonia	Necropsy: DAD, PF	Corticosteroids	NA	Death
2009 Muneoka Japan	M/82	Colorectal cancer	10	850	IP	NA	Corticosteroids	No	Complete resolution
2008 Arevalo Lobera Spain	F/73	Colorectal cancer	4	340	PF	Necropsy: DAD	Corticosteroids	No	Death
2008 Arevalo Lobera Spain	M/71	Colorectal cancer	4	340	ILD	Necropsy: interstitial inflammatory infiltrate, PF, bilateral bronchopneumonia	Corticosteroids Cyclophosphamide Immune globulins	Yes	Death
2009 Pena Alvarez Spain	M/62	Colorectal cancer	7	700	ILD	NA	Corticosteroids	No (family refused ICU admission)	Death
2009 Pena Alvarez Spain	M/77	Colorectal cancer	7	700	ILD	NA	Corticosteroids	No	Symptomatic resolution
2012 Hannan Australia	M/74	Colorectal cancer	6	600	ILD	Necropsy: DAD	Corticosteroids	No (patient refused intubation)	Death
2010 Han Lim Korea	M/64	Gastric cancer	8	680	ILD/PF	NA	Corticosteroids	Yes	Death
2013 Shogbon USA	F/78	Colorectal cancer	2	170	COP	NA	Corticosteroids	No	Improvement
2010 Shimura Japan	M/56	Colorectal cancer	6	510	ILD	NA	Corticosteroids	NA	Improvement
2010 Shimura Japan	M/71	Colorectal cancer	13	1105	ILD	NA	Corticosteroids	NA	Death
2010 Shimura Japan	M/73	Colorectal cancer	3	255	ILD	NA	Corticosteroids	NA	Improvement
2010 Shimura Japan	M/76	Colorectal cancer	5	425	ILD	NA	Corticosteroids	NA	Improvement
2010 Shimura Japan	M/73	Colorectal cancer	10	850	ILD	NA	Corticosteroids	NA	Death
2011 Joo Lee Korea	M/57	Colorectal cancer	8	680	ILD	LB: organizing pneumonia	Corticosteroids	No	Improvement
2009 Ohori Japan	M/69	Colorectal cancer	11	782	IP	NA	Corticosteroids	Yes	Complete resolution
2009 Ohori Japan	M/72	Colorectal cancer	11	935	IP	NA	Corticosteroids	No	Complete resolution
2012 Prochilo Italy	M/61	Gastric cancer	3	255	IP	NA	Corticosteroids	No	Improvement
2011 Watkins USA	M/69	Colorectal cancer	11	795	EP	Necropsy: DAD	Corticosteroids	Yes	Death
2011 Ishizone Japan	M/74	Colorectal cancer	22	1716	IP	NA	Corticosteroids	Yes	Death
2014 Cheong Soon UK	F/54	Colorectal cancer	12	1200	ILD	NA	Corticosteroids	No	Improvement
2011 Ryu en Jung Korea	M/55	Colorectal cancer	13	1105	IP	NA	Corticosteroids	Yes	Death
2011 Ryu Jung Korea	M/73	Colorectal cancer	8	680	IP	NA	Corticosteroids	Yes	Death
2011 Homma Japan	M/79	Colorectal cancer	12	1020	COP	NA	Corticosteroids	No	Improvement
2008 Piccolo Australia	M/70	Colorectal cancer	11	1100	IP	NA	Corticosteroids Cyclophosphamide	No	Improvement
2008 Piccolo Australia	M/62	Colorectal cancer	12	1200	IP	NA	Corticosteroids	No	Improvement
2010 Park Korea	M/76	Colorectal cancer	10	1000	ILD	NA	None	No	Stable disease
2014 Basyigit Turkey	M/60	Colorectal cancer	6	510	IP	NA	None	No	Improvement
2009 Dahlqvist Belgium	F/74	Colorectal cancer	12	1020	ILD	BAL: alveolitis TBB: BOOP	Corticosteroids	No	Improvement
2014 Hoon Choi Korea	F/76	Colorectal cancer	8	800	Sarcoidosis	NA	Corticosteroids	No	Improvement
2012 Pontes Brazil	M/75	Gastric cancer	9	765	ILD	BAL: no malignant cells	Corticosteroids	Yes	Death
2012 Pontes Brazil	M/64	Colorectal cancer	12	1020	ILD	LB: DAD, inflammatory infiltrate, diffuse thickening of interalveolar septa	Corticosteroids	Yes	Death
2013 Wildner Germany	M/62	Colorectal cancer	1	85	Infectious pneumonia	LB: granulomatous inflammation	Corticosteroids	Yes	Complete resolution
2015 De Weerdt Belgium	F/49	Colorectal cancer	6	580	ILD	LB: IP	Acetylcysteine Corticosteroids Immune globulins Cyclophosphamide	Yes	Complete resolution

*ARDS* Adult Respiratory Distress Syndrome, *BOOP* Bronchiolitis Obliterans Organizing Pneumonia, *COP* Cryptogenic Organizing Pneumonitis, *DAD* Diffuse Alveolar Damage, *EP* Eosinophilic Pneumonia, *ILD* Interstitial Lung Disease, *IP* Interstitial Pneumonia, *LB* Lung Biopsy, *OX* Oxaliplatin, *PF* Pulmonary Fibrosis, *TBB* Transbronchial Biopsy, *NA* Not Available

An overview of all of the reported FOLFOX-related pulmonary disease cases (Table 1) revealed that there seems to be an overwhelming male preponderance (male (M) 35/45 = 77,8%; female (F) 10/45 = 22,2%). Although the overall incidence of digestive cancer is higher in men [40–42], the incidence of oxaliplatin-related non-pulmonary toxic symptoms (e.g., neurotoxicity) is higher in women [43], which thus leaves the question of a possible increase in male gender-related pulmonary toxicity unresolved.

The mean age of all patients who developed pulmonary toxicity was 67.6 years (Y), with mean ages of 68.7 Y for the males and 63.9 Y for the women.

On average, ILD attributable to FOLFOX occurred after a median of 8 cycles of FOLFOX therapy (range 1–22 cycles) and a mean dose of 729.8 mg/m^2^ OX (range 85–1200 mg/m^2^, median 700 mg/m^2^). The men who developed pulmonary toxicity did so after a mean of 8.2 therapy cycles (range 1–22 cycles, median 8 cycles) and a mean OX dose of 738.3 mg/m^2^ (range 85–1716 mg/m^2^, median 732.5 mg/m^2^). The women who developed ILD did so after a mean of 7.6 cycles (range 2–12 cycles, median 7 cycles) and a mean OX dose of 701 mg/m^2^ (range 170–1200 mg/m^2^, median 630 mg/m^2^). These differences in mean age, number of cycles and mean dose were not significant between the men and women (all Ps > 0.5).

Five of the 45 patients (11.1%) with evidence of ILD on imaging studies received no other therapy other than the discontinuation of FOLFOX. These patients only exhibited mild symptoms (Table 2). None of these patients died. The other 40 patients (40/45 = 88.9%) with more marked symptoms were treated with corticosteroids, and 36 (36/40 = 90%) of these patients were treated with corticosteroids as a monotherapy (36/45 = 80% of the total study population). Twenty of the 36 patients treated with corticosteroids as monotherapy (55.6%) exhibited improvement, and 16 (44.4%) of these patients died.

**Table 2:** Clinical characteristics of patients with FOLFOX therapy related pulmonary disease treated with discontinuation of chemotherapy

Reference	Patient Gender/Age	Preexisting lung disease with the exception of lung metastasis	Smoking	Symptomes	0xygen supplement	Pulmonary function tests
Gagnadoux	F/60	None	No	Cough, progressive dyspnea on exertion	None	FEV1 1.980 L (90%) TLC 4.207 L (90%) DL_CO_ 4.4 ml/mmHg/min (59%)
Ruiz Casado	M/67	CT: some signs of pulmonary fibrosis in the basal portions	NA	None	None	NA
Wilcox	F/77	None	No	Dry cough, dyspnea on exertion	None	Normal lung volumes DL_CO_ 9.3 ml/mmHg/min (54%)
Park	M/76	Bronchial asthma	Ex-smoker 40 pack years	General systemic weakness, loss of appetite	None	FVC 2.26 L (95%) FEV 1 1.46 L (92%) DL_CO_ 10,8 ml/min/mmHg (109%)
Basyigit	M/60	None	Ex-smoker 14 pack years	Dyspnea on exertion	None	NA

*FEV1* Forced Expiratory Volume In One Second, *FVC* Forced Vital Capacity, *TLC* Total Lung Capacity, *DL*_*CO*_ Diffusing Capacity of carbon monoxide, *NA* Not Available

In the group of patients who exhibited improvement after treatment with corticosteroids as monotherapy, the mean number of FOLFOX therapy cycles was 6.95 (range 1–12 cycles, median 7 cycles). These patients had been treated with a mean OX dose of 630.35 mg/m^2^ (range 85–1200 mg/m^2^, median 690 mg/m^2^). Despite the fact that steroids have a much shorter half life in women than in men [44], five of the 7 female patients (71.4%) who were treated with corticosteroids as monotherapy exhibited improvement as opposed to only 15 of the 29 male patients (51.7%). This difference might be explained by the fact that females exhibit a greater sensitivity to corticosteroids [44].

The patients who died in this group had received a mean 9.56 cycles of therapy (range 4–22, median 8.5 cycles) and a mean OX dose of 812.25 mg/m^2^ (median 732.5 mg/m^2^).

Three of the 4 (75%) patients who were treated with a multimodal therapy regimen exhibited improvement. One patient was treated with a combination of corticosteroids and acetylcysteine, another was treated with a combination of corticosteroids and cyclophosphamide. One patient who was treated with a combination of corticosteroids, cyclophosphamide and immune globulins died. Prior to the FOLFOX treatment, he was diagnosed with Wegener’s disease with lung involvement, which probably contributed to the severity of the progression of ILD and his demise [10]. Our own patient was treated with a combination of corticosteroids, acetylcysteine, immune globulins and cyclophosphamide and exhibited improvement.

Information regarding intubation or not was available for 38 of the 45 patients (84.4%). Twenty-five of these patients did not receive mechanical ventilation (25/38 = 65.8%): 23 of them did not require it (23/25 = 92%), two of them were not intubated due to patient or family refusal.

The patients who were intubated had been treated with a mean OX dose of 742.92 mg/m^2^ (range 85–1716 mg/m^2^, median 680 mg/m^2^). The patients who were not intubated had been treated with a mean OX dose of 740.83 mg/m^2^ (range 100–1200 mg/m^2^, median 750 mg/m^2^).

Ten of the 13 patients (10/13 = 76.9%) who were intubated died. They had been treated with a mean OX dose of 821.10 mg/m^2^ (range 340–1716 mg/m^2^, median 722.5 mg/m^2^). The intubated patients who survived had received a mean OX dose of 482.33 mg/m^2^ (range 85–782 mg/m^2^, median 580 mg/m^2^).

Nine of the 10 intubated patients who died (90%) were treated with corticosteroids as monotherapy. The tenth intubated patient who died was the one with Wegener’s disease.

Two of the intubated patients who survived (2/3 = 66.67%) were treated with corticosteroids as monotherapy. The third patient who survived was our patient and was treated with acetylcysteine, corticosteroids, immune globulins and cyclophosphamide. All of the patients who had been intubated and survived developed complete resolution of their respiratory symptoms.

Seventeen out of the 45 (37.8%) patients died. The patients who died had been treated (mean of 9.24 episodes) with a mean dose of OX dose of 784. 47 mg/m^2^ (range 340–1716 mg/m^2^, median 700 mg/m^2^). The patients who survived had received a mean OX dose of 695.44 mg/m^2^ (range 85–1200 mg/m^2^, median 700 mg/m^2^) over a mean of 7 therapy cycles. Again, these differences were not significant.

In summary, we found that the discontinuation of the precipitating drug resulted in a 100% regression of the symptoms in the patients whose respiration was not too strongly affected, and we demonstrated that intubation heralded death in the majority of cases even with corticosteroid treatment.

## Discussion

Interstitial lung disease has diverse origins (e.g., autoimmune or systemic disease, exposure to drugs or herbs, infection, radiation, inhaled organic and inorganic substances, the late phase of the adult respiratory distress syndrome, cryptogenic) [45, 46] and often leads to death.

The diagnosis of a drug-induced lung disorder is considered when diagnostic algorithms have excluded all other potential aetiologies and when a distinct temporal association between exposure to the drug(s) and the development of respiratory complaints can be established [47]. In our patient, the respiratory symptoms arose after the 6th chemotherapy session, were rapidly progressive in nature, and were without microbial, autoimmune or environmental explanation. No infectious causes triggering the clinical picture were identified, although the high neutrophil count in the bronchoalveolar lavage fluid was initially strongly suggestive of microbial disease. In retrospect, we found that this finding was also compatible with drug-induced lung disease [20]. In view of the fact that an autoimmune disease could not be diagnosed, the administration of FOLFOX was considered to be the probable and most plausible cause of the biopsy-proven interstitial pneumonitis. Similar histological findings have been reported in three other cases of FOLFOX-related ILD [10, 32, 39].

To which of the three components of the FOLFOX regimen should the development of ILD be attributed? Thus far, there have been no reports linking folinic acid (leucovorin) in monotherapy to pulmonary toxicity. Five-fluorouracil is a thymidilate synthase inhibitor whose antimetabolite properties are enhanced by folates. It is one of the most frequently used chemotherapeutic agents and is applied in mono- and combined therapy for various solid malignancies of the head, neck, breast, lungs, gastrointestinal tract, prostate and bladder. Known side effects include alopecia, stomatitis, emesis, coronary spasms, hand-foot syndrome, diarrhoea and myelosuppression [48, 49]**.** There has only been one (Japanese) report of pulmonary toxicity accompanying the administration of 5-FU as monotherapy [50]. Oxaliplatin is a third-generation platinum derivative (diaminocyclohexane, containing platinum) that blocks DNA replication and transcription through the induction of intrastrand or interstrand lesions and DNA protein cross links [51]. Oxaliplatin is active against breast, gastric and colon cancers, renal cell carcinoma, sarcoma and cisplatin-resistant cell lines and tumour models including lung, ovarian, cervix, colon and leukaemia cell lines [51]. Known side effects include alopecia, peripheral sensory neuropathy (limb dysesthesia or paresthesia), haematological abnormalities (anaemia, thrombocytopenia, neutropenia), electrolyte disturbances (hyponatraemia, kalaemia), hepatocellular injury, nausea and vomiting, ototoxicity and laryngeal dysesthesia [6, 52, 53]. An important indirect argument pointing in the direction of oxaliplatin as “the” pulmonary toxicity-inducing culprit lies in the observation that respiratory symptoms present during FOLFOX therapy do not recur after rechallenge with a 5-FU- and leucovorin-containing but oxaliplatin-deprived chemotherapy cocktail [4, 7, 12, 14, 16–19].

When ILD is thought to be secondary to a chemical insult, discontinuation of the causative agent should be the first therapeutic intervention. Although sufficient for some, not all patients will experience improvement or full recovery after the cessation of the culprit compound. In our patient, the FOLFOX administration had already been discontinued, and acetylcysteine (supplying glutathione) was administered from day one in our hospital. Given that arguments linking oxaliplatin administration to glutathione depletion exist [30, 32], it seemed logical to continue glutathione supplementation because this molecule plays an important role in protecting the lungs against oxidative damage, which is a possible and probable contributing factor to the emergence of interstitial pneumonitis and subsequent evolution to pulmonary fibrosis.

Accounting for the severity of the illness, corticosteroid therapy, which is a well-established therapy modality, was initiated shortly after disease onset. Because no favourable respiratory evolution over a 14-day steroid course was observed, and no autoimmune disease had been diagnosed in the meantime, intravenous immune globulins (IVIgs) were given in an attempt to reduce the deposition of excessive amounts of extracellular proteins (particularly collagen-I) in the lung and thus prohibit the progression of the lung fibrosis already observed in the patient’s lung biopsy. These IVIgs were administered over a five-day period. The rationale for this treatment was found in experimental data that indicated that IVIgs are capable of preventing and treating bleomycin-induced pulmonary fibrosis in mice through the reduced expression of collagen-I protein in the affected lungs [23, 24]. Postulated mechanisms of this anti-fibrotic action of IVIg include modulation of cytokine production, inhibition of the complement reaction and inhibition of the CD95 receptor (Fas) activity through the presence of anti-Fas antibodies in IVIg [23, 54, 55]. Subsequently, one dose of cyclophosphamide, a nitrogen mustard alkylating and lymphocyte-modulating agent, was administered. This immunosuppressive steroid-sparing agent is used to treat autoimmune inflammatory disorders and associated interstitial lung disease and is frequently added to corticosteroid therapy to enhance the clinical response due to the additional suppression of immunoreactions that cause lung damage [56]. In our patient, an intravenous pulse was administered because at that moment, an unspecified immunological process (possibly with vasculitis) leading to pulmonary fibrosis seemed possible.

In our analysis of the 45 reported cases of FOLFOX-related pulmonary disease, we found that the administered treatments were variable in terms of the drugs used and highly variable in terms of the durations and dosages of the corticosteroids used. Hence, it was impossible to determine the exact contribution of each individual drug or drug dosage to the recoveries of the patients. Moreover, in some patients, ILD regression was observed without special treatment but “merely” after discontinuation of FOLFOX. The fact that our patient exhibited no improvement after the withdrawal of the causative agent and developed a full clinical and radiographic recovery after the introduction of acetylcysteine, corticosteroids, immune globulins and cyclophosphamide, led us to believe that a causal relationship between this multimodal therapy and respiratory progress exists.

Considering the frequency with which FOLFOX therapy is used worldwide, the reported incidence of ILD seems extremely low. We wondered why this is. Are not all cases of FOLFOX related pulmonary toxicity reported, or are only a small number of people genetically or otherwise predisposed? Are there age, gender, ethnic or geographical differences in the concentrations of innate NO, glutathione or profibrotic agents (e.g., type 2 CD4-positive lymphocytes, CD40 receptor and ligand interactions, interleukin-4, interleukin-10, interleukin–13, Th3-type cytokine-transforming growth factor beta 1, and platelet-derived growth factor) [22–24] that contribute to the development of pulmonary toxicity? Could there be interindividual differences in oxaliplatin metabolism that lead to toxicity? Indeed, the biotransformation of oxaliplatin leads to the formation of aquated platinum forms in the blood. Three compounds can be found: total platinum, free (ultrafiltrable) platinum, and erythrocyte platinum [57]. Platinum is rapidly cleared from the plasma by renal elimination. However, what if the erythrocyte platinum is not as harmless as generally accepted but rather exhibits toxic effects in isolated cases, and what if the clearance of erythrocyte platinum is delayed in a minority of patients?

## Conclusions

FOLFOX therapy related pulmonary toxicity is uncommon but often lethal in respiratory insufficient patients. We urge oncologists and critical care physicians not to limit their interventions to the discontinuation of chemotherapy, artificial ventilation, corticosteroid therapy and glutathione replenishment and to consider the gradual introduction of additional immune-modulating agents (e.g., immune globulins and cyclophosphamide) whenever life-threatening respiratory symptoms in oxaliplatin-treated patients do not subside.

## Abbreviations

5 – FU: 5-fluorouracil
BAL: Bronchoalveolar lavage
CRP: C-reactive protein
CT: Computed tomography
ICU: Intensive care unit
ILD: Interstitial lung disease
IP: Interstitial pneumonia
IVIg: Intravenous immune globuline
OX: Oxaliplatin
PCR: Polymerase chain reaction
PEEP: Positive end expiratory pressure
